# *Kras* activation in endometrial organoids drives cellular transformation and epithelial-mesenchymal transition

**DOI:** 10.1038/s41389-021-00337-8

**Published:** 2021-06-25

**Authors:** Yoshiaki Maru, Naotake Tanaka, Yasutoshi Tatsumi, Yuki Nakamura, Makiko Itami, Yoshitaka Hippo

**Affiliations:** 1 Department of Molecular Carcinogenesis, Chiba Cancer Center Research Institute, Chiba, Japan; 2 Department of Gynecology, Chiba Cancer Center, Chiba, Japan; 3 Division of Oncogenomics, Chiba Cancer Center Research Institute, Chiba, Japan; 4 Division of Surgical Pathology, Chiba Cancer Center, Chiba, Japan

**Keywords:** Endometrial cancer, Cancer stem cells

## Abstract

*KRAS*, an oncogene, is frequently activated by mutations in many cancers. *Kras*-driven adenocarcinoma development in the lung, pancreas, and biliary tract has been extensively studied using gene targeting in mice. By taking the organoid- and allograft-based genetic approach to these organs, essentially the same results as in vivo models were obtained in terms of tumor development. To verify the applicability of this approach to other organs, we investigated whether the combination of *Kras* activation and *Pten* inactivation, which gives rise to endometrial tumors in mice, could transform murine endometrial organoids in the subcutis of immunodeficient mice. We found that in *Kras*^*G12D*^-expressing endometrial organoids, *Pten* knockdown did not confer tumorigenicity, but *Cdkn2a* knockdown or *Trp53* deletion led to the development of carcinosarcoma (CS), a rare, aggressive tumor comprising both carcinoma and sarcoma. Although they originated from epithelial cells, some CS cells expressed both epithelial and mesenchymal markers. Upon inoculation in immunodeficient mice, tumor-derived round organoids developed carcinoma or CS, whereas spindle-shaped organoids formed monophasic sarcoma only, suggesting an irreversible epithelial-mesenchymal transition during the transformation of endometrial cells and progression. As commonly observed in mutant *Kras*-driven tumors, the deletion of the wild-type *Kras* allele was identified in most induced tumors, whereas some epithelial cells in CS-derived organoids were unexpectedly negative for *Kras*^*G12D*^. Collectively, we showed that the oncogenic potential of *Kras*^*G12D*^ and the histological features of derived tumors are context-dependent and varies according to the organ type and experimental settings. Our findings provide novel insights into the mechanisms underlying tissue-specific *Kras-*driven tumorigenesis.

## Introduction

Ras is a small guanosine-5’-triphosphate (GTP)-binding protein that transmits external stimuli to downstream signaling pathways, such as Raf, PI3K, and Ral-GEF. These effectors orchestrate various vital cellular processes, including gene transcription, cell proliferation, acquisition of cell motility, and inhibition of cell death [1]. Although Ras activation is normally regulated through GTP-binding and degradation in a transient and reversible manner, its constitutive activation is often observed in various types of cancer, mostly by missense mutations in the GTP-binding sites at codons 12, 13, and 61 or by gene amplification [2]. Epithelial malignancies have preferences for *KRAS* mutations among the three *RAS* genes, namely *HRAS, NRAS*, and *KRAS*; although *KRAS* mutation rate varies among tissues, it can be as high as ~90% in pancreatic cancer; 20–50% in carcinomas of the colon, lung, and hepatobiliary tract; and much less frequent in other carcinomas [3]. Using genetically engineered mice (GEM) models, many studies have demonstrated the causal role of the *Kras*^*G12D*^ mutation in tumor development, through cooperation with other common genetic aberrations in each cancer type [4]. Thus, *Kras*^*G12D*^ has been established as a *bona fide* oncogene.

Endometrial cancer (EC) is the most common gynecological malignancy and the fourth most common female cancer in industrial countries [5,6,]. EC is typically classified into two types based on clinical and histological characteristics. Type 1, comprising 80% of all ECs, is associated with estrogen dependency, endometrial hyperplasia, and a favorable prognosis. The predominant histological subtype is endometrioid carcinoma, harboring frequent mutations in *PTEN* (52–78%), *PIK3CA* (36–52%), *KRAS* (15–43%), *ARID1A* (25–48%), and *CTNNB1* (23–24%). In contrast, type 2 is associated with estrogen independency, endometrial atrophy, and poor prognosis, and comprises various minor subtypes including serous carcinoma, clear cell carcinoma, and carcinosarcoma (CS), with high mutation rates in *TP53* (60–91%) [7,8,]. Not in a mutually exclusive manner with these recurrent mutations, microsatellite instability (MSI) is found in 40% of endometrioid carsinoma and 2% in serous carcinoma, thereby leading to the proposal of a novel classification based on the mutational burden; the four categories included *POLE*-mutated (ultramutated), MSI (hypermutated), copy-number low, and copy-number high, the first two of which have significantly better prognosis [7].

Because *PTEN* has the highest mutation rate across EC, the effect of its inactivation has been extensively investigated using GEM. Although *Pten*^−/−^ mice died during embryogenesis [9], all *Pten*^*+/−*^ mice developed precancerous lesions, 25% of which eventually developed EC within a year [10]. *PR-Cre; Pten*^*flox/flox*^ mice, in which *Pten* was deleted by the Cre-recombinase induced in a uterine-specific manner, frequently developed invasive EC by 3 months of age [11]. After the local injection of adenovirus *Cre* into the uterus of *Pten*^*flox/flox*^ mice, EC development was observed, albeit with partial penetrance [12,13,]. Concurrent *Kras*^*G12D*^ accelerated *Pten*-dependent EC development [14,15,], suggesting that *Kras*^*G12D*^ plays an oncogenic role in the endometrium. Alternatively, GEM with genitourinary tract-selective *Trp53* deletion developed type 2 EC in 84% of mice at 58–68 weeks of age [16]. These findings suggest that the tumorigenic potential of endometrial cells and the histological features of EC may be genetically determined to a considerable extent, although more studies are required to fully elucidate its pathogenesis.

Organoid culture is an emerging technology that enables the long-term propagation of normal epithelial cells in a physiological setting [17,18,]. It is a three-dimensional culture method using the Matrigel, extracellular matrix that mimics the basement membrane, and serum-free medium supplemented with stem cell niche factors for each tissue. Owing to the high utility, its application has been expanding to many research fields, including infectious diseases [19], developmental biology [20], and tissue regeneration [21]. We reconstituted a combination of common genetic aberrations in organoids with lentiviral vectors encoding short hairpin RNA (shRNA) and *Cre*, which were inoculated into nude mice. Even without a tissue-specific microenvironment, certain combinations of genetic aberrations that are particularly common in certain cancers robustly give rise to subcutaneous tumors. For example, *Apc* inactivation and *Kras* activation in the intestinal organoids markedly accelerated tumorigenesis caused by *Apc* knockdown alone [22]. In organoids from the lung [23], hepatobiliary tract [24,25,], and pancreas [26], the development of full-blown tumors was achieved not by *Kras* activation alone, but by the concurrent inactivation of the p53 or Rb pathway. Since these outcomes are consistent with the results of earlier GEM studies [22,23,25,26,], we hypothesized that such organoid and allograft-based approach can establish an alternative carcinogenesis model in any organ.

To verify this notion, we investigated whether organoids from the murine endometrium could be transformed by reconstitution of genetic aberrations that developed type 1 EC in GEM. Through examination of transduced organoids, subcutaneous tumors, and derived organoids, we gained novel insights into multi-layered interactions that are involved in tumor development.

## Results

### *Pten* knockdown allowed endometrial organoids to propagate after lentiviral infection

**Fig. 1:**
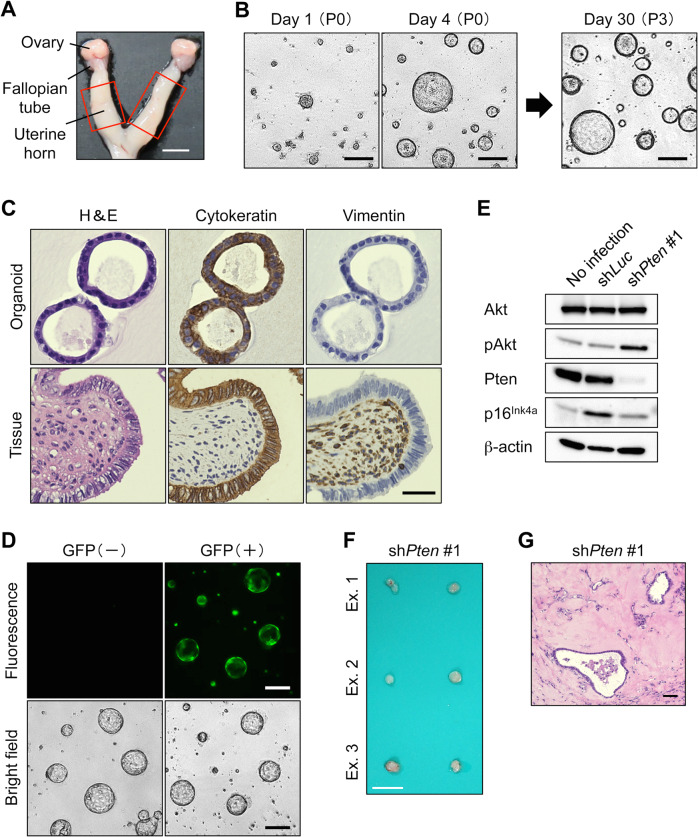
Endometrial organoids with *Pten* knockdown exhibited low tumorigenic potential. **A** Macroscopic view of the gynecologic tract. A portion of the uterine horn (*red rectangle*) was dissected. Scale bar, 2.5 mm. **B** Representative images of endometrial organoids. Time-lapse images of primary (P0) cells and after 3 passages (P3) are shown. Scale bar, 200 μm. **C** Immunohistochemical (IHC) staining. *Upper panel*, organoids. *Lower panel*, uterine tissue. Immunostaining results of serial sections with antibodies against cytokeratin or vimentin are shown. Scale bar, 30 μm. **D** Lentiviral gene transduction of endometrial organoids. The same views of *GFP*-transduced organoids are shown. Scale bar, 200 μm. **E** Western blot analysis of transduced organoids. Both *Pten* knockdown and Akt phosphorylation were achieved by sh*Pten*. The induction of p16^Ink4a^ is prominent following lentiviral transduction with sh*Luc*, but not with sh*Pten*. **F** Macroscopic findings of an isolated nodule. *Pten* knockdown alone did not result in the tumorigenic potential of endometrial organoids. Scale bar, 10 mm. **G** Histological findings of nodules derived from organoids with *Pten* knockdown. A few glands with mild atypia are observed after H&E staining. Scale bar, 50 μm.

To conduct an organoid-based tumorigenicity assay, we isolated and propagated primary epithelial cells from the murine uterine horn (Fig. 1A). Endometrial organoids that robustly propagated for at least a few months had rounded cystic structures, whereas stromal cells spontaneously disappeared within a few passages (Fig. 1B). Organoids consisted of columnar epithelial cells lined in a monolayer as verified by the cytokeratin^+^/vimentin^-^ staining pattern (Fig. 1C). Efficient gene transduction was verified using a lentivirus vector encoding GFP (Fig. 1D).

Considering that most previous GEM models for EC were generated by the ablation of *Pten* [9–13], we first evaluated the tumorigenic potential of the organoids with *Pten* knockdown. By introducing shRNAs against *Pten* (hereafter, sh*Pten*) into the endometrial organoids, *Pten* knockdown and the subsequent Akt activation were verified compared to the shRNA against *luciferase* (sh*Luc*) (Fig. 1E). Although organoids without infection or with sh*Pten* continued to proliferate, those with sh*Luc* failed to propagate after the first passage and had a considerable elevation of the cell cycle inhibitor p16^Ink4a^, suggesting the deleterious effects of lentiviral infection. Upon the inoculation of organoids with sh*Pten* into nude mice, only tiny nodules (Fig. 1F) containing a few glands with mild atypia of ductal or cystic shape developed (Fig. 1G). These observations suggest that despite the advantages of in vitro proliferation and unlike earlier in vivo studies, *Pten* knockdown alone is insufficient to induce tumorigenesis in endometrial organoids.

### Endometrial organoids with *Kras*^*G12D*^ and *Pten* knockdown did not develop tumors

**Fig. 2:**
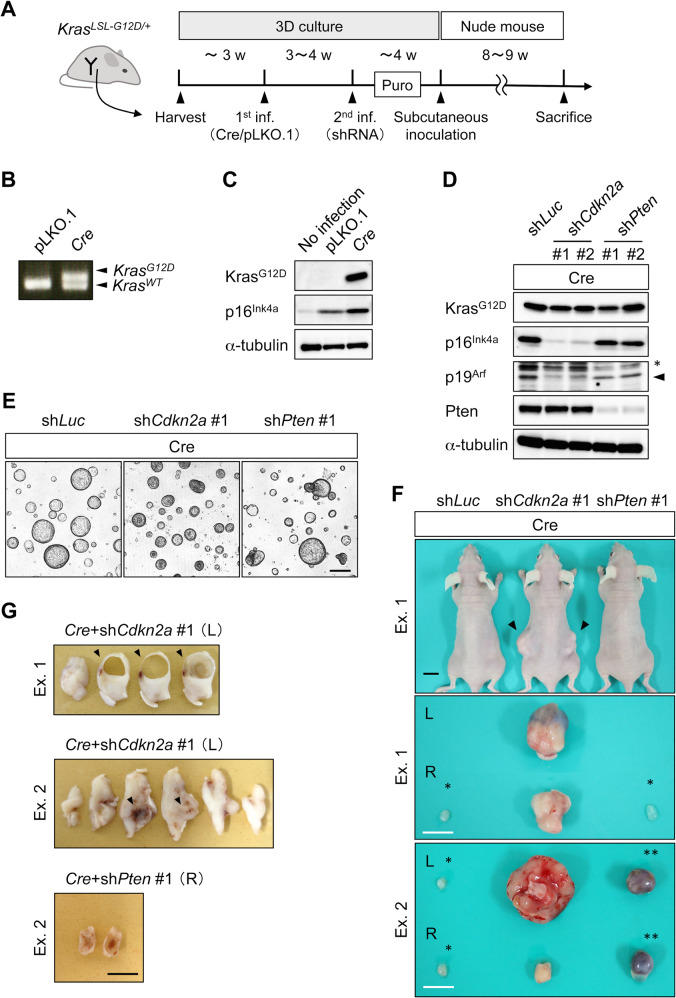
*Kras*^*G12D*^ organoids with *Cdkn2a* knockdown developed CS. **A** Two-step protocol for organoid generation from *Kras*^*LSL-G12D/+*^ mouse. **B** Genomic PCR analysis of transduced organoids. The upper band depicted as *Kras*^*G12D*^ indicates the successful recombination of the *Kras* locus by *Cre*-recombinase, whereas the lower band depicts an amplicon for the WT allele of *Kras*. **C** Western blotting analysis of endometrial organoids. Stepwise inductions of p16^Ink4a^ by infection itself and *Cre* transduction were observed. **D** Western blot analysis of Kras-activated endometrial organoids. The knockdown of the targeted gene products was achieved by the introduction sh*Cdkn2a* and sh*Pten* clones. sh*Cdkn2a* suppressed its two products p16^Ink4a^ and p19^Arf^. Non-specific bands (*) and specific bands (*arrowhead*) are shown for the p19^Arf^ panel. **E** Transduced organoids visualized under phase-contrast microscope. Scale bar, 200 μm. **F** Tumor development in nude mice. *Upper panel*, subcutaneous tumors (*arrowheads*). Scale bar, 10 mm. *Middle and lower panels*, resected tumors. Representative results from two independent experiments (Exs. 1 and 2) are shown. Matrigel plugs (*single asterisk*) and cystic lesions (*double asterisks*) are classified as non-tumorous lesions. Scale bar, 10 mm. **G** Macroscopic view of sliced tumors after formalin fixation. Scale bar, 10 mm. *Cre* + sh*Cdkn2a* #1: a solid tumor with cystic formation (*arrowhead*) in Ex. 1 and with necrosis (*arrowhead*) in Ex. 2. *Cre* + sh*Pten* #1: multilocular cyst in Ex. 2.

The activation of the Ras pathway has been implicated in human EC [7]. As *Kras*^*G12D*^ accelerated the *Pten*-dependent EC development in mice [14,15,], we investigated whether this combination of genetic alterations could drive tumor development in endometrial organoids. We applied a two-step gene transduction protocol, namely, introduction of *Cre* and shRNA transduction (Fig. 2A). Organoids from *Kras*^*LSL-G12D/+*^ mice were subjected to lentiviral infection, in which *Kras*^*G12D*^ is transcribed upon the *Cre*-mediated excision of the STOP codon flanked by the two LoxP sequences (LSL) [28]. Subsequently, we detected a newly emerged *Kras*^*G12D*^ amplicon slightly longer than the *Kras*^*WT*^ amplicon by a single LoxP (Fig. 2B) and observed the induction of the Kras^G12D^ protein (Fig. 2C), establishing successful recombination.

**Fig. 3:**
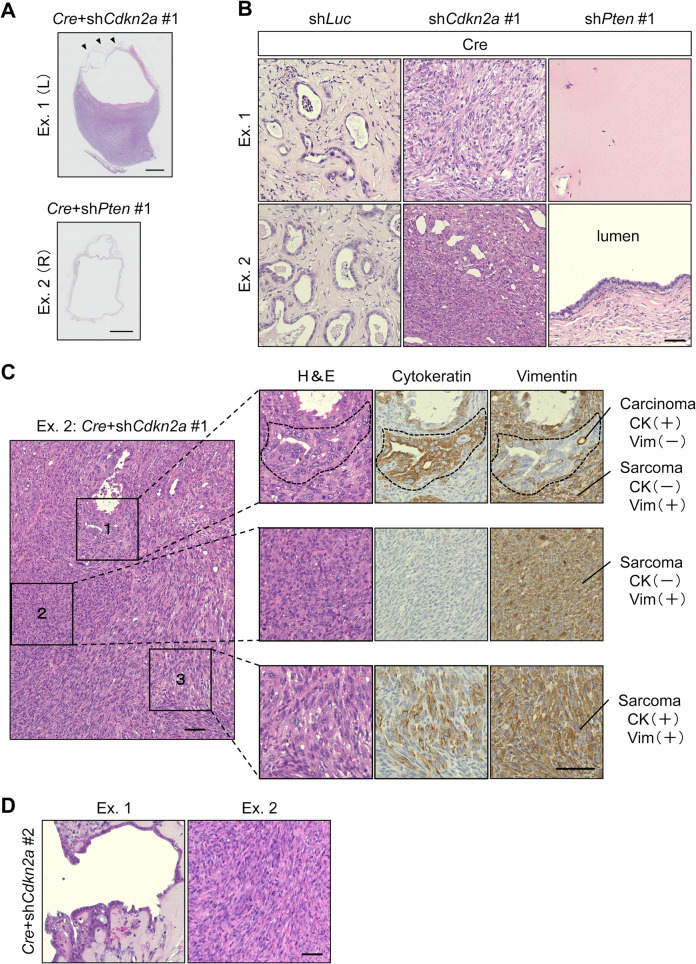
Histological features of organoid-derived CS and monophasic sarcoma. (A) H&E staining of thinly sliced sections. *Cre* + sh*Cdkn2a* #1 is a sarcoma with cyst (*arrowheads*), whereas *Cre* + sh*Pten* #2 is a multilocular cyst. Scale bar, 10 mm. (B) H&E staining of thinly sliced sections of subcutaneous nodules from Exs. 1 and 2. Matrigel plugs induced by *Cre* + sh*Luc* contained only a few glands with mild atypia. Tumors induced by *Cre* + sh*Cdkn2a* #1 are sarcoma in Ex. 1 and carcinosarcoma in Ex. 2. Matrigel plugs (Ex. 1) and that by *Cre* + sh*Pten* #1 is a cystic lesion (Ex. 2). Scale bar, 50 μm. (C) Characterization of heterogeneous lesions of carcinosarcoma. H&E and IHC staining against cytokeratin and vimentin of serial sections for areas 1–3 are shown. Magnified view of the corresponding areas is shown. Dotted lines in area 1 indicate cytokeratin^+^/vimentin^-^. Scale bar, 50 μm. (D) H&E staining of the tumors induced by *Cre* + sh*Cdkn2a* #2. Cystic lesion from Ex. 1 and sarcoma lesion from Ex. 2 are shown. Scale bar, 50 μm.

Although organoids with *Cre* steadily proliferated, those with the backbone vector pLKO.1 gradually stopped propagating over passages, similar to the organoids after transduction with sh*Luc*. Because p16^Ink4a^ was induced after lentiviral infection and more prominently after *Kras*^*G12D*^ induction, two potent shRNA clones against *Cdkn2a* encoding p16^Ink4a^ and p19^Arf^ were added to the second infection, along with sh*Pten* and sh*Luc*, to avoid cell cycle arrest (Fig. 2D). No morphological changes were observed (Fig. 2E). Most *Kras*^*G12D*^ organoids with sh*Luc* did not develop tumors in the nine cases tested (Fig. 2F), with the exception of one case of CS, which was defined by the presence of both carcinoma and sarcoma components in the tumor (Fig. S1, Table S1). *Kras*^*G12D*^ organoids with sh*Pten* clone #1 developed cysts in two out of three cases (Fig. 2F, G), in which the lumen was covered with a monolayer of columnar cells with mild atypia (Fig. 3A, B). Another sh*Pten* clone #2 did not induce tumors in two experiments, even after introduction into the *Kras*^*G12D*^ organoids that gave rise to sarcoma upon the introduction of sh*Luc* (Table S1, Fig. S1). These observations suggested the marginal tumorigenic potential of *Kras*^*G12D*^, which was not promoted by *Pten* knockdown.

### *Kras*^*G12D*^ organoids with *Cdkn2a* knockdown or *Trp53* deletion developed CS

In contrast, *Kras*^*G12D*^ organoids with sh*Cdkn2a* developed solid tumors (Fig. 2F) within eight weeks in all five cases (Table S1). The tumors partly contained cystic lesions or necrosis (Figs. 2G, 3A) and were predominantly occupied by spindle-like cells and partly by atypical glandular cells. Tiny nodules originating from the *Kras*^*G12D*^ organoids with sh*Luc* only contained a few glands with mild atypia (Fig. 3B). Among the five solid tumors, two were diagnosed with CS, two with monophasic sarcoma, and one with a combination of sarcoma and cyst (Table S1). In the CS cases, most carcinoma and sarcoma cells separately resided as cytokeratin^+^/vimentin^-^ and cytokeratin^-^/vimentin^+^ populations, respectively (Fig. 3C). However, a subset of sarcoma cells showed cytokeratin^+^/vimentin^+^ expression, indicating an intermediate state between typical carcinomas and sarcomas (Fig. 3C). This finding suggested an epithelial origin of the sarcoma cells. In the two experiments with another potent sh*Cdkn2a* clone, cysts or sarcomas were induced in *Kras*^*G12D*^ organoids (Fig. 3D), thus eliminating the possibility of off-target effects by shRNA. These results suggest that *Kras*^*G12D*^ expression and *Cdkn2a* knockdown cooperate for tumor development, preferentially toward the induction of sarcomatous differentiation.

**Fig. 4:**
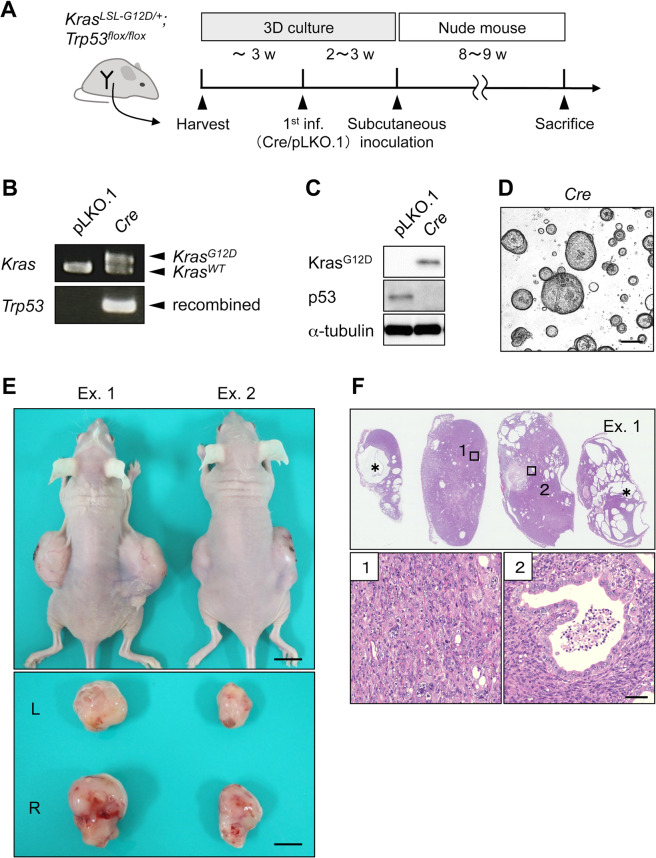
*Kras*^*G12D*^ organoids with deleted *Trp53* developed CS. **A** One-step protocol for organoid generation from *Kras*^*LSL-G12D/+*^*; Trp53*^*flox/flox*^ mice. **B** Genomic PCR analysis of endometrial organoids from *Kras*^*LSL-G12D/+*^*; Trp53*^*flox/flox*^ mice. The presence of the PCR products for the amplicons of *Kras*^*G12D*^ and the recombined allele of *Trp53* indicates the successful *Cre*-mediated recombination in both loci. **C** Western blot analysis of transduced organoids. Induction of *Kras*^G12D^ and loss of p53 at the protein level were demonstrated. **D** A representative image of endometrial organoids after *Kras* activation and *Trp53* deletion. Images for mock-infected organoids are not shown owing to the lack of steady propagation after lentiviral infection. Scale bar, 200 μm. **E** Tumor development following Kras activation and *Trp53* deletion. *Upper panel*, a representative image of subcutaneous tumors developed in nude mice. *Lower panels*, resected tumors. Representative results of two independent experiments (Exs. 1 and 2) are shown. Scale bar, 10 mm. **F** Histological findings in subcutaneous tumor. *Upper panel*, H&E staining of thin sections for the same four slices illustrating multiple cystic structures in the solid tumor (*asterisks*). *Lower panel*, Magnified images of the two areas (*boxes*) are shown. Note that the area 1 consists exclusively of spindle-like, pleomorphic cells, whereas area 2 contains carcinoma cells showing a glandular structure. Scale bar, 50 μm.

In human uterine CS, *TP53* is the most frequently mutated gene [29,30,]. Consistent with this finding, GEM with uterine-specific *Trp53* deletion developed CS after a long latency and only in ~10% of the tumors [16]. This suggested that other genetic alterations were required to accelerate CS development; therefore, we investigated whether *Kras*^*G12D*^ and p53 loss could synergize tumorigenesis. After *Cre* transduction of endometrial organoids from *Kras*^*LSL-G12D/+*^*; Trp53*^*flox/flox*^ mice (Fig. 4A), the simultaneous induction of *Kras*^*G12D*^ expression and *Trp53* deletion (Figs. 4B, 4C) was verified. We observed the steady proliferation of organoids (Fig. 4D), but not in organoids with pLKO.1 as predicted. Upon the inoculation into nude mice, solid tumors developed within eight weeks in all four cases (Fig. 4E). Notably, the tumors were invariably diagnosed as CS (Table S1, Fig. 4F). Thus, *Trp53* deletion also cooperated with *Kras*^*G12D*^ expression for CS development in endometrial organoids.

### Tumor-derived organoids retained epithelial-mesenchymal transition (EMT) state

**Fig. 5:**
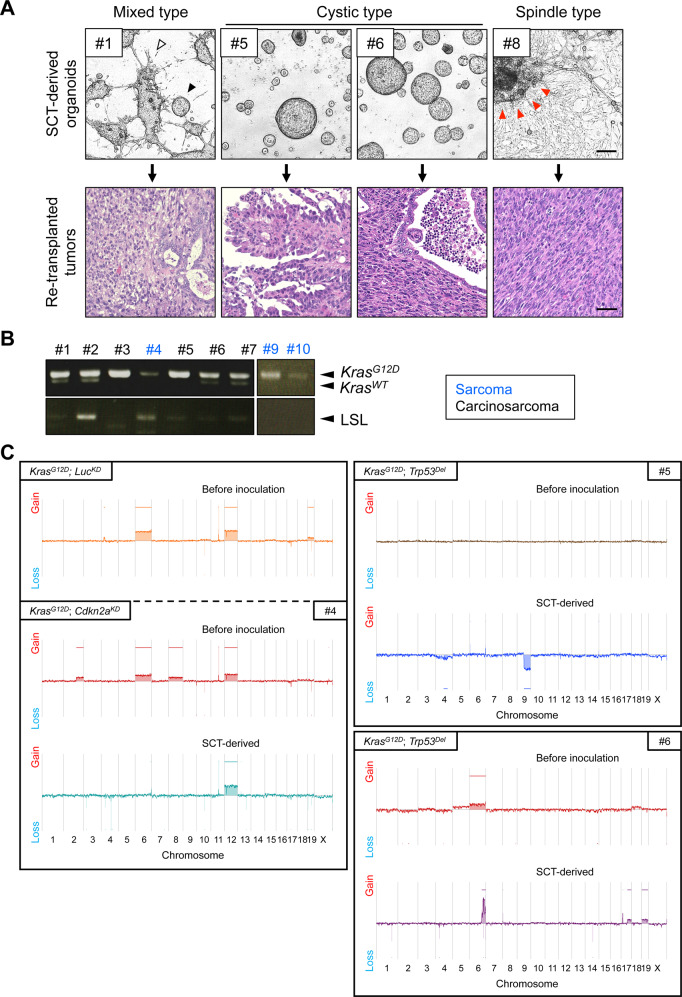
Morphological and genomic heterogeneity among tumor-derived organoids. **A** Morphological classification of organoids recovered from subcutaneous tumors. *Upper panel*, mixed type (#1) contained both spindle-like cells that protrude from the organoids (*white arrowhead*) and standard cystic organoids (*black arrowhead*). Cystic type (#5 and #6) contained only standard cystic organoids, whereas spindle-type (#8) contained only spindle-like cells with occasional cell aggregation (*red arrow heads*). Scale bar, 200 μm. *Lower panel*, H&E staining of tumors induced by the re-inoculation of subcutaneous tumor-derived organoids. Mixed type induced the formation of carcinosarcoma (CS) (#1); cystic type, adenocarcinoma (#5), or CS (#6); and spindle-type sarcoma (#8). Scale bar, 50 μm. **B** Genomic PCR analysis of the *Kras* locus in tumor-derived organoids. **C** Copy number alterations in transduced or tumor-derived organoids as identified by array CGH analysis. SCT, subcutaneous tumor; Luc^KD^, introducing sh*Luc*.

Regardless of the presence of genetic alterations, endometrial organoids stereotypically maintained their round cystic shape. In contrast, those recovered from subcutaneous CS or sarcoma displayed diverse morphological features ranging from cystic, spindle-like, and combined types (Fig. 5A). Previously, we revealed that nude mice-derived stromal cells could not survive the current serum-free culture conditions over several passages [25,26,]. The presence of spindle-like cells in six out of eleven tumor-derived organoids (TDOs) strongly suggests that their transformed nature was stably retained during recovery from subcutaneous tumors. In addition, one combined type and two spindle-like type TDOs developed CS and monophasic sarcoma, respectively, whereas two cystic-type TDOs developed monophasic carcinoma or CS (Fig. 5A, Table S2). These observations indicated that spindle-like cells might have originated from epithelial cells and that carcinoma cells could have undergone EMT to sarcoma in an irreversible manner.

### Tumor-derived organoids displayed heterogeneous statuses in the *Kras* loci

For pancreatic and biliary tract organoids, TDOs generated from organoid-based *Kras*-driven carcinogenesis models were completely negative for the LSL cassette in the conditional allele [25,26,]. Considering that a fraction of cells retained the LSL at the time of inoculation, this finding suggested that Kras activation was a definite requirement for adenocarcinoma development in these organoids. Similarly, we examined the *Kras* locus in nine endometrial TDOs using genomic PCR analysis (Fig. 5B, Table S2). The emergence of the *Kras*^*G12D*^ amplicon was detected in all TDOs as predicted, whereas the residual LSL cassette was unexpectedly detected in some cases (Fig. 5B, Table S2), indicating the survival of untransformed cells without *Kras*^*G12D*^ within the CS. In addition, the intensity of *Kras*^*WT*^ amplicon was clearly fainter than that of *Kras*^*G12D*^ in four cases and was undetectable in five cases (Fig. 5B, Table S2), suggesting the spontaneous deletion of the *Kras*^*WT*^ allele, as occasionally observed in the organoid-based pancreatic cancer model [26].

To evaluate the genetic instability comprehensively during CS development, we performed array CGH analysis on three pairs of transduced organoids and their corresponding TDOs. For *Kras*^*G12D*^ organoids with both *Cdkn2a* knockdown (#4) and *Trp53* deletion (#5, #6), the pre-inoculated organoids and TDOs exhibited genome stability, although no recurrent amplifications or deletions were detected (Fig. 5C). Thus, the *Kras* loci may have undergone unique alterations during the development of CS and sarcoma.

### Significant transcriptomal changes in organoids underlay tumorigenesis

**Fig. 6:**
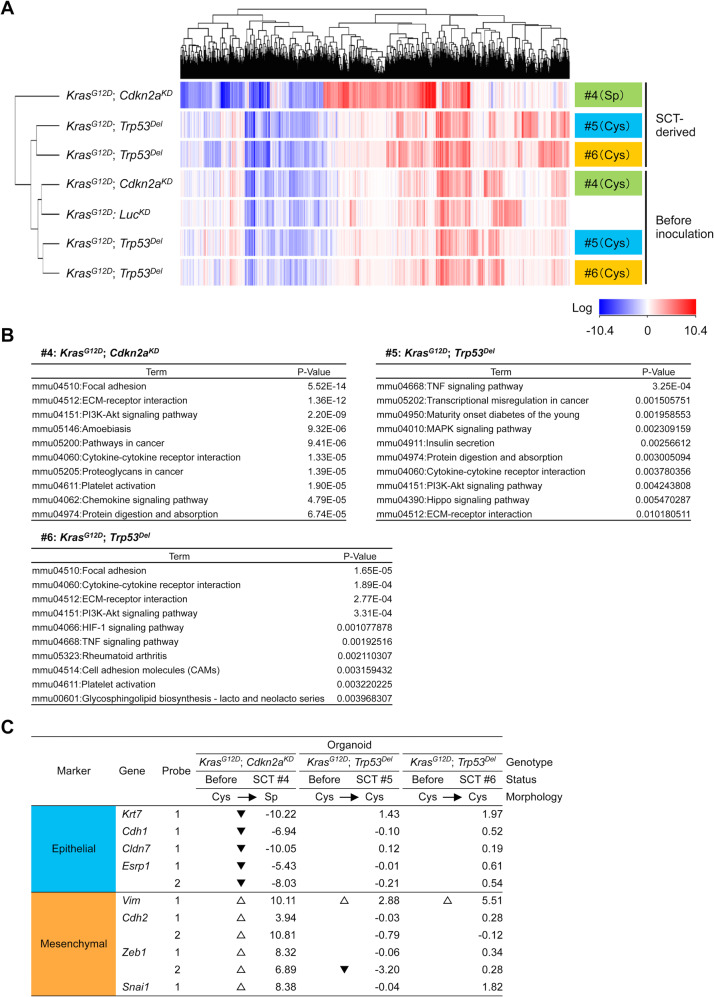
Transcriptomic changes in organoids after inoculation in nude mice. **A** Heat map showing differentially expressed genes in transduced or tumor-derived organoids as identified by using microarray analysis. Colors range from blue to red corresponding to genes with low and high expression, respectively. **B** Activated function was inferred from up-regulated genes in each tumor-derived organoid. **C** Gene expression of epithelial–mesenchymal transition markers in tumor-derived organoids.

To better characterize the pathogenesis of CS from organoids, we conducted a transcriptome analysis. Based on hierarchical cluster analysis, organoids before inoculation and TDOs (#5 and #6) were relatively similar, although a spindle-type TDO (#4) was observed as an outlier (Fig. 6A). The Kyoto Encyclopedia of Genes and Genomes database was used to identify signaling pathways that were significantly altered during tumorigenesis. Among the up-regulated gene functions, three out of the top ten were shared by all TDOs, including ECM-receptor interaction, the PI3K-AKT signaling pathway, and cytokine-cytokine receptor interaction (Fig. 6B). Considering that CS and sarcoma likely originated from epithelial organoids, we further examined the expression of EMT marker genes in TDOs. Spindle-type organoids (#4) showed high expression levels of several known mesenchymal markers, such as *Vimentin, Cdh2, Zeb1, and Snai1*, and low expression levels of epithelial markers, such as *Krt7, Cdh1, Cldn7, and Ersp1* [31] (Fig. 6C). In contrast, the transcriptome profile of cystic-type organoids (#5 and #6) was similar to that of pre-inoculated organoids, except for a moderate increase in *Vimentin* (Fig. 6C). These results indicate that gene expression profiles reflect the morphology of TDOs.

## Discussion

We previously showed high concordance of tumorigenicity caused by certain combinations of genetic aberrations between GEM and organoid-based carcinogenesis models [22,25,26,]. Regarding *Pten*-driven tumorigenicity in the uterus, tumor development was not demonstrated in this study, even in the presence of *Kras*^*G12D*^. One possible explanation for the observed discrepancy is that the uterine-specific gene targeting in mice by *PR-Cre* mice [11] or the local injection of adeno-*Cre* [12,13,] may in fact have resulted in gene recombination in both epithelial and stromal cells of the uterus. In line with this notion, mice with uterine epithelial-specific ablation of *Pten* did not develop EC [32]. Considering that the resultant *Pten* knockdown in the stroma is not common in sporadic cases of human EC, the overestimated tumorigenic potential could be attributed to the interplay between the epithelia and the stroma of the uterus. Similar observations were previously documented in intestinal tumorigenesis, wherein *Pten* deletion in both the stroma and epithelia in mice led to tumor development, which was not observed when deletion was specific to the epithelium [33,34,] Another possibility is that even normal uterine stroma could play a pro-tumorigenic role. In a cell-based study, the introduction of *myr-Akt* or sh*Pten* into primary murine endometrial cells, followed by co-transplantation with the uterine stroma from neonatal WT mice, resulted in the development of adenocarcinoma in the kidney capsule [35], suggesting the importance of the microenvironment of the uterine stroma in EC development. Lastly, given that the MSI subtype of human EC preferentially harbors mutations in *KRAS* and *PTEN* [7], it is tempting to speculate that additional inactivation of mismatch repair genes in endometrial organoids could achieve type I EC development, which is worthy of further investigations.

Another unique property of endometrial organoids is the propagation arrest associated with lentiviral infection, which has never been observed in any gastroenterological organoids under almost the same culture conditions [22–26]. Considering the infinite propagation of uninfected organoids and p16^Ink4a^ induction following lentiviral infection, endometrial organoids may be particularly sensitive to DNA damage caused by viral genomic integration. Although the underlying mechanisms remain elusive, this finding prompted us to knockdown *Cdkn2a* in endometrial organoids, which unexpectedly led to the *Kras*-dependent development of CS or sarcoma. In human EC cases, *KRAS* mutations are rarely found in serous carcinoma (3%) but are more prevalent in endometrioid carcinoma (24%) and CS (12%); *TP53* mutations are significantly more common in serous carcinoma (88%) and CS (91%) than in endometrioid carcinoma (21%) [36]. These findings are consistent with CS development in endometrial organoids upon Kras activation and p53 loss in this study. In GEM with identical genetic alterations, CS development was also observed in the ovary [37], while adenocarcinoma was always induced in the pancreatobiliary system for both in vivo and ex vivo [25,26,38,39,]. These findings suggest that gynecological organs may be predisposed to CS due to some inherent epigenetic regulation.

We further demonstrated the development of CS from epithelial cells in two ways: from transduced cystic organoids and from cystic-type TDOs originating from organoid-derived CS. In addition, we identified a fraction of CS cells that expressed both epithelial and mesenchymal markers. These findings are consistent with the notion that uterine CS is predominantly a monoclonal neoplasm of epithelial origin, rather than generated by the collision of two distinct cell populations [40,41,]. This indicates irreversible EMT during transformation and progression from carcinoma to sarcoma. Single-cell analysis at each step of this model may help clarify the molecular events involved in CS development. Further investigation on whether our results can be extrapolated to in vivo GEM is warranted. Also, our study might provide a unique resource for CS research, in which models are still few.

CS induced in this study was “homologous”, which by definition demonstrates the presence of spindle-like sarcoma cells resembling inherent uterine stroma, such as fibroblasts and smooth muscle cells. In contrast, the uterine epithelial-specific ablation of *Fbxw7* and *Pten* in mice resulted in the development of “heterologous” CS, having a sarcoma component of differentiation toward bone and cartilage in half of the cohorts in three months [42]. Interestingly, mutations in *Trp53* and *Kras* were found in four and one cases, respectively, among 76 late-stage heterologous CS cases, suggesting the relevance of the deregulation in these two genes in the common pathway to the development of uterine CS. In line with this notion, tumors with concurrent mutation in *KRAS* and *TP53* distributed across all four subtypes of uterine CS, which respectively correspond to the same four categories in EC [43]. We speculate that certain genetic alterations may determine the differentiation of cell lineages and molecular subtypes of CS, although further investigation is required.

The characterization of the *Kras* locus in organoids and TDOs revealed several aspects of CS pathogenesis. First, the copy number of the *Kras*^*WT*^ allele frequently decreased in TDOs. We have previously documented the complete loss of the *Kras*^*WT*^ allele, albeit at a low frequency, in *Kras*^*G12D*^-expressing TDOs originating from pancreatic organoids [26]. The frequent loss of *KRAS*^*WT*^ has also been reported in tumors with mutant *KRAS* in humans [44]. Collectively, these findings are consistent with the notion that *Kras*^*WT*^ acts as a relative tumor-suppressor gene in the presence of oncogenic *Kras* and that its deletion leads to the hyperactivation of the Kras pathway [45]. Second, the complete loss of *Kras*^*WT*^ was observed in the TDOs. Because this is unlikely to occur spontaneously in normal cells, this observation negates the possibility that host-derived stromal cells can transform into CS cells. Last, the presence of residual cells without recombination in the *Kras* locus was revealed in TDOs. This finding is contrary to previous observations that TDOs originating from adenocarcinoma comprises only cells expressing *Kras*^*G12D*^, but not those that retain *Kras*^*WT*^, thus highlighting the critical roles of Kras activation in the development of adenocarcinoma [25,26,]. Consequently, based on this unexpected finding, we speculated that uterine CS cells might support the survival of normal epithelial cells to retain *Kras*^WT^, which might subsequently promote the proliferation of CS cells.

In conclusion, we established uterine CS using an organoid-based approach. Although patient-derived organoids (PDOs) for normal and cancer tissues in gynecologic organs have been recently established [27, 46–49], to the best of our knowledge, there are almost no reports on PDOs from CS cases. Therefore, our generated tumor organoids will be useful in preclinical studies of CS. In addition, future comparisons among organoids derived from ex vivo carcinogenesis models, GEM, and PDOs can provide further insights into the mechanisms underlying endometrial tumorigenesis.

## Materials and methods

### Mice studies

Female mice of the C57BL/6 J strain and Balb/cA^*nu/nu*^ (nude mice) at 5 weeks of age were purchased from CLEA Japan Inc. (Tokyo, Japan). Conditional knock-in mice heterozygous for the Lox-STOP-Lox-KrasG12D allele (hereafter referred to as *Kras*^*LSL-G12D/+*^ mice) and *Trp53*^*flox/flox*^ mice were obtained from the Jackson Laboratory (Bar Harbor, ME, USA) and maintained in a C57BL/6 J background. These mice were intercrossed to generate *Kras*^*LSL-G12D/+*^*; Trp53*^*flox/flox*^ mice. Genotyping was performed after weaning as previously described [28,50,]. Animal studies were carried out with the approval of the Chiba Cancer Center for Ethics in Animal Experimentation.

### Isolation and organoid culture of endometrial cells

Bilateral uterine horns were isolated from C57BL/6 J mice at approximately 10 weeks of age. The tissues were minced into 2–3 mm pieces, washed several times with cold PBS, and dissociated with 2 U/mL dispase II and 1 mg/mL collagenase P (Roche Diagnostics K.K., Tokyo, Japan) for 30 min at 37 °C. Dissociated cells were subjected to the Matrigel bilayer organoid culture (MBOC) protocol [22]. Briefly, cells were resuspended in the medium supplemented with L-glutamine solution (Wako, Osaka, Japan), penicillin/streptomycin (Sigma-Aldrich, St. Louis, MO), amphotericin B suspension (Wako), 50 ng/mL EGF (Peprotech, Rocky Hill, NJ), 250 ng/mL R-spondin1 (R&D, Minneapolis, MN), 100 ng/mL Noggin (Peprotech), 10 μM Y27632 (Wako, Osaka, Japan), 1 μM Jagged-1 (AnaSpec, Fremont, CA), and 2.5 μM CHIR-99021 (Cayman Chemical, Ann Arbor, MI), and plated on 65 μL of solidified Matrigel (BD Biosciences, Franklin Lakes, NJ) per well in a 12-well plate. After overnight incubation at 37 °C, floating or dead cells were removed, leaving only viable cells attached onto Matrigel, which were covered with 70 μL of Matrigel and the medium. Passage was conducted every 5–10 days at 1:2 to 1:3 dilution. In each passage, organoids were dissociated using Accutase (Innovative Cell Technologies, San Diego, CA) for 5 min at 37 °C and vigorous pipetting, followed by seeding on Matrigel.

### Lentiviral vectors and infection

Lentiviral particles were generated using the ViraPower Lentiviral Expression System (Invitrogen, Carlsbad, CA) following the manufacturer’s instructions. The collected viral supernatants were passed through a 0.45-µm filter, concentrated 10-fold with PEG-it Virus Precipitation Solution (System Biosciences), and stored at −80 °C until further use. The lentiviral transduction of organoids was performed as previously described [51]. Puromycin selection (3 µg/mL) was conducted for 4–5 days. For shRNA transduction, pLKO.1-puro vectors (Sigma-Aldrich, St. Louis, MO) targeting murine *Cdkn2a* (TRCN222731 and TRCN231227) and *Pten* (TRCN322421 and TRCN28992) were used. As a negative control shRNA, sh*Luciferase* (SHC007) was used. LV-*Cre* pLKO.1 (Addgene plasmid 25997) [52] was used to remove the sequence flanked by the two LoxP sequences. *Cre*-mediated recombination was verified by genomic PCR as previously described [28,50,]. pCDH-CMV-MCS-EF1-copGFP (System Biosciences, Mountain View, CA) was used to monitor transduction efficiency.

### Tumorigenicity assays in nude mice

Transduced endometrial cells were propagated as organoids in Matrigel. Organoids corresponding to 5 × 10^5^ cells were resuspended in 200 μL of medium mixed with Matrigel at a 1:1 ratio and inoculated into the dorsal skin of nude mice. After 8–9 weeks, palpable tumors or residual Matrigel plugs from the injected sites were isolated for histological examination or cell culture. The tumors were processed essentially in the same way as cell preparation for the primary organoid culture. As serum-free culture media do not support the survival of tumor-derived stromal cells, a pure population of epithelial cells is normally obtained within a few passages. In some cases, tumor-derived organoids were re-implanted into the dorsal skin.

### Western blotting

Organoids were harvested after Cell Recovery Solution (BD Biosciences) treatment for 1 h on ice and lysed using RIPA buffer (50 mM Tris-HCl at pH 7.4 supplemented with 150 mM NaCl, 1% NP-40, 0.5% sodium deoxycholate, 0.1% SDS, and 1 mM EDTA) supplemented with 1 M NaF, 0.1 M Na_3_VO_4_, 1 M βGP, and a protease inhibitor cocktail (Nacalai Tesque, Kyoto, Japan). Protein concentrations were quantified using a Pierce BCA protein assay kit (Thermo Fisher Scientific, Waltham, MA), and 5–10 µg of protein was separated under reducing conditions in regular tris-glycine buffer on a gradient gel, SuperSep Ace 5 20% (Wako). The proteins were transferred onto Immobilon-P PVDF membranes (Sigma-Aldrich) using semi-dry electrophoresis. The membrane was blocked with PBS-T containing 5% non-fat dry milk at 4 °C overnight, followed by incubation with primary antibodies for 90 min at room temperature. The antibodies used were as follows: RasG12D (#14429, Cell Signaling Technology, Danverse, MA, 1:1,000), p16^Ink4a^ (sc-1207; Santa Cruz Biotechnology, Dallas, TX, 1:2,000), Pten (#9559, Cell Signaling Technology, 1:2,000), p53 (#2524, Cell Signaling Technology, 1:2,000), p19^Arf^ (NB200-174, Novus Biologicals, Centennial, CO, 1:1,000), Akt (#4961, Cell Signaling Technology, 1:2,000), pAkt (#4060, Cell Signaling Technology, 1:2,000), α-tubulin (T5168, Sigma-Aldrich, 1:20,000), and β-actin (013–24553, Wako, 1:2,000). The membranes were incubated with the corresponding secondary antibodies (mouse: NA931, GE Healthcare, Buchinghamshire, UK, rabbit: 7074, Cell Signaling Technology) for 45 min at room temperature and visualized using ImmunoStar LD (Wako). Chemiluminescent images were captured using an ImageQuant LAS 4000 mini (GE Healthcare).

### Histopathological analysis

All tumors were fixed in 10–20% buffered neutral formalin, dehydrated, and embedded in paraffin. For the organoids, Matrigel was lysed by treatment with Cell Recovery Solution (BD Biosciences) and embedded in iPGell (GenoStaff, Tokyo, Japan) before fixation. The formalin-fixed, paraffin-embedded samples were sliced into 3-µm-thick sections and subjected to hematoxylin and eosin (H&E) and immunohistochemical staining with the following antibodies: pan cytokeratin clone AE1/AE3 (ab27988, Abcam, Tokyo, Japan, 1:40) and vimentin clone EPR3776 (#2707-1, Epitomics, Burlingame, CA, 1:500). The reactions were visualized using the Dako REAL EnVision Detection System (Dako, Glostrup, Denmark) with diaminobenzidine chromogen as the substrate.

### Array-based comparative genomic hybridization analysis

To extract genomic DNA from organoids, a NucleoSpin Tissue Kit (Takara, Shiga, Japan) was used. DNA quantity and quality was assessed using a NanoDrop spectrophotometer and agarose gel electrophoresis (Thermo Fisher Scientific), respectively. A SureTaq DNA Labeling Kit (5190-03399, Agilent, Santa Clara, CA) was used to chemically label 500 ng of genomic DNA with either ULS-Cy5 or ULS-Cy3 dye. Hybridization with labeled DNA was performed using a SurePrint G3 mouse CGH microarray 4 × 180 K (G4826A, Agilent). Scanning and image analysis were performed using Agilent Feature Extraction ver.11.0 (Agilent) and a SureScan Microarray Scanner (G4900DA, Agilent). Agilent Genomic Workbench ver.7.0.4.0 software was used to visualize, detect, and analyze chromosomal patterns in the microarray profiles. Organoids without lentiviral infection were analyzed as reference for estimating copy number variations.

### Transcriptome analysis

Total RNA was extracted using an RNeasy Mini Kit (Qiagen). The RNA quality and quantity of each sample were checked and measured, respectively, using an Agilent 2100 Bioanalyzer (Agilent). All samples had an RNA integrity number greater than 7.8. The cRNA was prepared from 200 ng of total RNA using a Low Input Quick Amp Labeling Kit, one-color (5190-2305, Agilent), and labeled with cyanin3. Hybridization was performed using the SurePrint G3 Mouse Gene Exp v2 Array Kit 8 × 60 K (G4852B, Agilent). Scanning and image analysis were performed on a SureScan Microarray Scanner (G4900DA, Agilent). Microarray data were analyzed using GeneSpring GX ver.13.1 software (Agilent). Organoids without lentiviral infection served as reference. Data are available at the GEO database (GSE175512).

## Supplementary information

Supplementary information
