# Supplementary material for: Kras activation in endometrial organoids drives cellular transformation and epithelial-mesenchymal transition
Source: Oncogenesis. 2021 Jun 25;10(6):46. doi: 10.1038/s41389-021-00337-8 (PMC8233399; doi:10.1038/s41389-021-00337-8)
Supplement: Supplementary file 1 — Supplementary information [file 41389_2021_337_MOESM1_ESM.pdf]

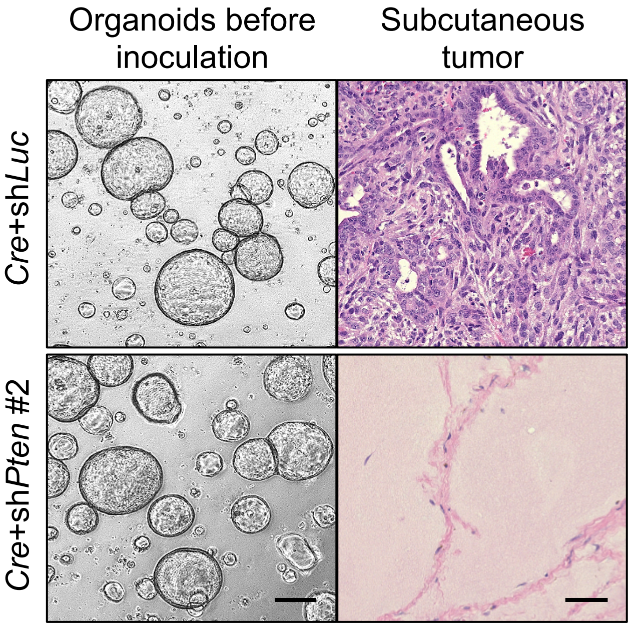

Figure S1. Occasional induction of CS upon Kras activation.
Left panel, phase-contrast microscopy images of transduced organoids from *Kras*$^{LSL\text{-}G12D/+}$ mice before inoculation. Scale bar, 200 μm. *Right panel*, H&E staining of thinly sliced sections of tumors or nodules induced by *Cre*+sh*Luc* or *Cre*+sh*Pten* #2, respectively. Scale bar, 50 μm.

Table S1. Summary of subcutaneous tumor development in endometrial organoids.

Genotype of organoids	Lentiviral infection		Pathological diagnosis of tumors					Total
	First	Second	None	Cy	S	S+Cy	CS	
WT	sh*Luc**	–	–	–	–	–	–	3
	sh*Pten* #1	–	3	0	0	0	0	3
Kras$^{LSL\text{-}G12D/+}$	pLKO.1*	–	–	–	–	–	–	4
	Cre	sh*Luc*	8	0	0	0	1	9
	Cre	sh*Cdkn2a* #1	0	0	2	1	2	5
	Cre	sh*Cdkn2a* #2	0	1	1	0	0	2
	Cre	sh*Pten* #1	1	2	0	0	0	3
	Cre	sh*Pten* #2	2	0	0	0	0	2
Kras$^{LSL\text{-}G12D/+}$;Trp53$^{flox/flox}$	pLKO.1*	–	–	–	–	–	–	4
	Cre	–	0	0	0	0	4	4
							Total	39

*Unable to proceed to second infection or inoculation due to failure in propagation.

None, no nodule or normal glands with no atypia; S, sarcoma; CS, carcinosarcoma; Cy, cyst.

Table S2. Characterization of subcutaneous tumors and organoids.

Case No.	#1	#2	#3	#4	#5	#6	#7	#8	#9	#10	#11
Genotype of organoids	KL	KC	KC	KC	K5	K5	K5	K5	KC	KC	KC
First SCT	CS	CS	CS	S+Cy	CS	CS	CS	CS	S	S	S
SCT-derived organoid	Mix	Cy	Sp	Sp	Cy	Cy	Mix	Sp	Sp	Sp	Sp
Kras^{G12D} allele	+	+	+	+	+	+	+	*n.t.*	+	+	*n.t.*
Kras^{WT} allele	±	±	−	−	−	±	±	*n.t.*	−	−	*n.t.*
LSL cassette	±	+	−	+	±	±	±	*n.t.*	−	−	*n.t.*
Second SCT	CS	*n.t.*	*n.t.*	S	C	CS	*n.t.*	S	*n.t.*	*n.t.*	*n.t.*

SCT, subcutaneous tumors

first SCT, subcutaneous tumors developed by inoculation of transduced organoids.

second SCT, SCT developed by re-transplantation of first SCT-derived organoids

KL, *Kras*$^{LSL\text{-}G12D/+}$; (LV-*Cre* + sh*Luc*).

KC, *Kras*$^{LSL\text{-}G12D/+}$; (LV-*Cre* + sh*Cdkn2a*).

K5, *Kras*$^{LSL\text{-}G12D/+}$; *Trp53*$^{\text{flox/flox}}$; (LV-*Cre*)

CS, carcinosarcoma. S, sarcoma. Cy, cyst.

Mixed, mixed type. Cy, cystic type. Sp, spindle-type.

n.t., not tested.
